# Study on the Pore and Microstructure Fractal Characteristics of Alkali-Activated Coal Gangue-Slag Mortars

**DOI:** 10.3390/ma13112442

**Published:** 2020-05-27

**Authors:** Hongqiang Ma, Jianwei Sun, Chao Wu, Cheng Yi, Yu Li

**Affiliations:** 1 School of Mechanics and Civil Engineering, China University of Mining and Technology (Beijing), Beijing 100083, China; hongqiang.ma@canterbury.ac.nz; 2 Department of Civil and Natural Resources Engineering, University of Canterbury, Christchurch 8041, New Zealand; yu.li@pg.canterbury.ac.nz; 3 Department of Civil Engineering, Tsinghua University, Beijing 100084, China; jianwei2019@mail.tsinghua.edu.cn; 4 School of Transportation Science and Engineering, Beihang University, Beijing 100191, China

**Keywords:** alkali-activated coal gangue-slag, compressive strength, microstructure, fractal dimension, pore characteristic parameters

## Abstract

Just as it is regarding ordinary cement-based materials, the pore structure and microstructure of alkali-activated materials are disordered. It is essential to predict the macroscopic properties by studying the pore and microstructure fractal characteristics of materials. In this paper, the effects of slag content and alkali activator modulus on compressive strength, porosity, and microstructure of alkali-activated coal gangue-slag (AACGS) mortar were studied. Further, with the help of mercury intrusion porosimetry (MIP) data and the MATLAB programming, the pore and SEM photos fractal dimensions of AACGS mortar specimens were obtained, respectively, and the relationship between the microscopic fractal dimensions and the macroscopic strength and the structural characteristics of pores was established. The results show that the pore fractal dimension has a good linear relationship with the compressive strength and pore characteristic parameters (porosity, total pore area, and average pore diameter, etc.). With the increase of slag content, the SEM photos fractal dimension of AACGS mortar specimens increases, and the fractal dimension and compressive strength also show a significant positive linear relationship. The two fractal characterization methods can be used in the alkali-activated material system and have important guiding significance for predicting the macroscopic strength and pore characteristic parameters of the material.

## 1. Introduction

In recent years, research on alkali-activated materials has shown a rapid growth trend. Researchers have been more and more favored alkali-activated materials due to excellent early mechanical properties [1], excellent resistance to chemical erosion [2], and fire resistance [3]. A large number of studies have shown that the commonly used raw materials for alkali-activated include slag, fly ash, metakaolin [4,5,6], and some researchers have also applied lime [7], red mud [8], coal gangue [9,10,11], and rice husk ash [12], and the research of alkali-activated binary materials is more and more extensive. With the advantages of low energy consumption and low environmental impact, alkali-activated materials are expected to become the most promising materials to replace cement cementitious materials.

Coal gangue is the mining solid waste discharged in the process of driving, mining, and coal washing. China’s coal mining volume ranks first in the world, resulting in coal gangue becoming the most massive industrial solid waste in China. The main chemical components of coal gangue are SiO_2_ and Al_2_O_3_, and the activity of raw coal gangue is very deficient; it shows pozzolanic activity after calcination at 700 °C, which has similar chemical characteristics with fly ash and metakaolin. Slag belongs to the material of high calcium pozzolanic material, while coal gangue belongs to the material of low-calcium pozzolanic material. The high SiO_2_ and Al_2_O_3_ in the coal gangue are mixed with the high CaO in the slag to obtain the better alkali-activated binary coal gangue-slag materials [13,14].

The microstructural properties of alkali-activated materials depend on their microscopic structural characteristics. The phase systems of alkali-activated binary and multiple materials are multi-layered, and the phase components of various structures are combined according to their respective interface properties. The changes in mechanical and durability properties vary with the changes in the internal microstructure. The macro-mechanics and micro-scale of materials are related to the internal structure of materials, and the heterogeneity of mixed materials increases the complexity of the internal structure of materials [15]. For alkali-activated pure pulp material, qualitative or quantitative tests can by means of X-ray diffraction (XRD), Fourier transform infrared spectroscopy (FT-IR), thermal gravity–differential thermal gravity (TG-DTG), and nuclear magnetic resonance (NMR) (TG-DTG, XRD, FT-IR, NMR test specimens are powder). However, in view of the alkali-activated mortar or concrete that can be used in the characterization, this means the mesoscopic level contains mercury intrusion method (MIP), scanning electron microscope (SEM), and gas adsorption. The data obtained from the SEM photos are qualitative and cannot quantitatively characterize the changing characteristics and trends of the internal structure.

The fractal theory is a branch of nonlinear continuity and has scale independence (representational fractal is invariant under usual geometric transformations). Liu et al. [16] applied the fractal theory to metakaolin concrete and found that the fractal method can quantitatively analyze the characteristics of the internal microstructure changes in metakaolin concrete. In the study of concrete subjects, the fractal theory has been applied successfully to cementitious materials, pores, and fracture properties, etc., showing its fractal characteristics [17,18]. Furthermore, the research of fractal features based on surface features and pore structure measurement techniques is increasingly favored by researchers [19,20]. However, different scholars give different fractal theories and measurement methods, but they can combine microscopic analysis dimensions with the macroscopic performance [21,22]. The calcined coal gangue material is different from the commonly used materials such as slag, fly ash, and silica fume; however, the fractal theory research on coal gangue material has not been widely studied.

In this paper, the effects of slag content and alkali activator modulus on the compressive strength and microstructure of alkali-activated coal gangue-slag (AACGS) mortar will be investigated, and the surface morphology and microstructure of mortar specimens will be observed by means of MIP method and SEM. The fractal dimension of each specimen was calculated to explore the relationship between the pore fractal dimension and the compressive strength, pore characteristic parameters (obtained by the MIP method). The SEM image counting box dimension of each specimen was calculated based on Matlab programming to achieve quantitative characterization of the microstructure and explore the relationship between the fractal dimension of the SEM image and compressive strength. This paper provides an analytical method to study the internal laws between microstructure and macroscopic physical and mechanical properties of advanced concrete materials.

## 2. Microstructure Fractal Feature Theory Review

In recent years, researchers have explored the fractal theory to characterize the fractal characteristics of cement paste and obtained specific achievements and concerns [23,24]. At present, the fractal theory research on the fractal characteristics of building materials with the help of surface laser scanning (SLS) [25], scanning electron microscopy (SEM) [26], and mercury intrusion porosimetry (MIP) [27] and other measurement technologies are increasingly favored by researchers. Fractal dimension is an essential parameter in fractal characterization, which describes the degree of complexity and irregularity of complex systems.

### 2.1. Calculation of Microstructure Fractal Dimension

The fractal theory can be traced back to the fractal geometry founded by Professor B.B. Mandelbort. Winslow [28] first applied the fractal theory to the fractal characteristics of cement microstructure. Fractal dimension is a parameter characterizing the complexity of the fractal body. Common dimension expressions include Hausdorff dimension [29], Box dimension [30], Similarity dimension [31], Correlation dimension [32], and Spectral dimension [33], etc. In 1994, Lange and Jennings [34] combined fractal theory with image analysis technology to study the pore structure of concrete and realized the combination of fractal theory and image analysis technology for the first time. Wittmann [35] applied the fractal theory to the micromechanics of concrete and gave macroscale (10^−1^–10^−3^ m), mesoscale (10^−3^–10^−4^ m), and microscale (10^−4^–10^−8^ m), three kinds of concrete structure characteristics, regarding concrete as a three-phase mixture composed of aggregate, cement, and interface. In the research of building materials, the commonly used fractal dimension measurement methods are the box dimension method and the yardstick method [36]. The values of the two fractal dimensions will be explained below.

I. Box dimension method. Assume that the F set is a non-empty bounded set in the Rn domain, and Nε(F) is the number of sets with a maximum diameter ε required to cover the F set. Nε(F) changes with the change of the maximum diameter. As a curve of lnNε(F)–lnε, the opposite number of the slope of the curve is the fractal box dimension value [36]. The box dimension is determined by the coverage of the same shape set. In order to quantitatively describe the internal microscopic changes of AACGS mortar under different slag content and different alkali activation modulus, the relationship between the box dimension and box quantity of SEM photos can be obtained by importing SEM photos into the computer program. After binarization processing of SEM photos, the fractal dimension program is used to calculate the fractal dimension. The opposite number of the slope of the curve is the fractal dimension value. In other words, the box dimension method is adopted in this paper to calculate the fractal dimension of SEM photos, which includes two steps: Binarization processing of SEM photos and fractal dimension numerical calculation.
II. Yardstick method. The complicated curve is decomposed by unit length, in which one end of the curve is used as the starting point and a circle of unit length r is drawn with the starting point as the center. The point that intersects the curve with the starting point is connected and the intersection point is used as the new starting point to repeat the circle. The total number of measured line segments is recorded as p(r) and the unit length r is changed; p(r) will also change. The specific functional relationship can be expressed as:
  p(r) = L/r∝r^−D^ (1)
  L∝r^1−D^ (2)
  where L represents the length of the fractal curve. As an lnL–lnr curve, 1−D represents the slope of the straight line, which is the value of the fractal dimension.

The straight lines and curves in the two-dimensional plane are unique. If the micro-image is regarded as a two-dimensional plane {(x, y)}, x and y represent the position of the pixel in the two-dimensional plane, and the dimensional value of the fractal curve is obtained, and its dimensional value is between 1–2. At present, there are few studies on fractal curves. If the micro-image is regarded as a three-dimensional space {(x, y, z)}, an uneven surface is formed on the basis of a two-dimensional plane, and its fractal dimension value is between 2–3 [37]. However, in the high-magnification SEM image, the SEM image presents the surface topography information of the specimen, which is a two-dimensional image. The area represented by the micro-image is limited, so it may be more accurate to use a two-dimensional plane for analysis. Therefore, this paper calculates the fractal dimension of the research object based on the grayscale SEM image. The binarization image of the SEM image can be obtained based on computer image processing and uses the box dimension method to calculate the fractal dimension of the binary image.

### 2.2. Calculation of Fractal Dimension in Mercury Intrusion Porosimetry

SEM method and MIP method have become the most commonly used microstructure testing methods due to their fast and straightforward test procedures, and SEM photos can be used to calculate the box dimension with the help of the computer. Similarly, the pore fractal dimension can also be obtained based on MIP test data. Typical pore structure fractal models include the Sierpinski gasket fractal model, the Menger sponge fractal model, the Von Koch curve, and the Atzeni fractal model [38]. 

The pore fractal dimension of the specimen can be determined using volume measurement. Song et al. [39] calculated by constructing a volume fractal model, decomposing a cube with side length R into multiple small cubes with side length r, removing some small cubes according to a specific rule and the remaining small cubes were N_l_(m). The remaining small cubes are continuously divided and discarded, and the size of the remaining cubes reduced continuously, and the number increased. After k operations, the size of the remaining small cubes r_k_ can be expressed as:

r_k_ = R/m^k^ (3)

The number of small cubes remaining is:

(4) $$N_{k}\;=\;N_{1}^{k}\;=\;{\left(r_{k}/R\right)}^{-D}$$

where D is the fractal dimension of the pore volume, which can be expressed as D = logN_1_/logm.

The pore volume V_k_ can be expressed as:

(5) $$V_{k}\propto r_{k}^{3-D}$$

Assuming k→∞ or 0, it can be expressed as:

dV/dr ∝r^2−D^ (6)

It can get according to the pore volume:

lg[−dV_p_/dr]∝(2−D)lg(r) (7)

Based on the fractal dimension calculation method of pore volume and the principle of the MIP, the pore volume V_p_ can measure by the mercury volume V injected under a certain pressure P, and r is the corresponding pore diameter. According to the existing research, there are two methods to calculate the pore fractal dimension based on MIP data:

I. Take the log of dV/dP and dP in the MIP method data and draw the dV/dP–dP curve; the fractal dimension of the pore surface according to the slope of the curve is calculated.
II. Take the log of dV/dr and dr in the MIP method data and draw the dV/dr–dr curve; the fractal dimension of the pore volume according to the slope of the curve is calculated.

## 3. Experimental and Test Methods

### 3.1. Raw Materials and Specimen Preparation

**Figure 1:**
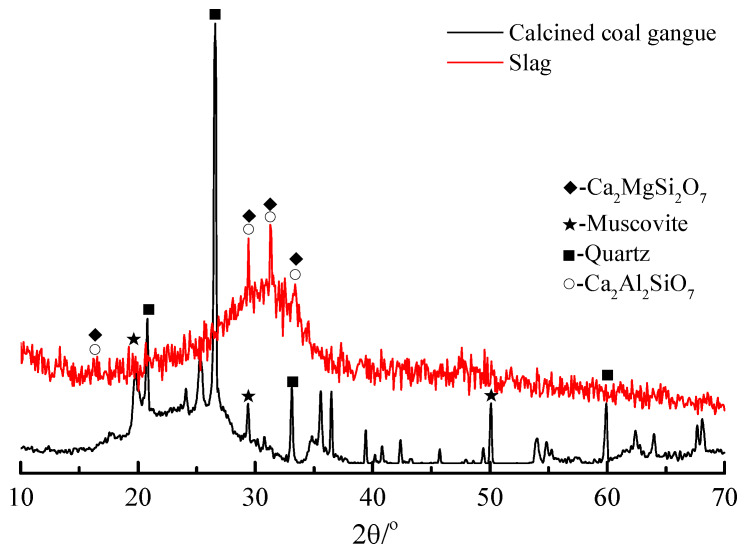
XRD pattern of calcined coal gangue and slag.

**Figure 2:**
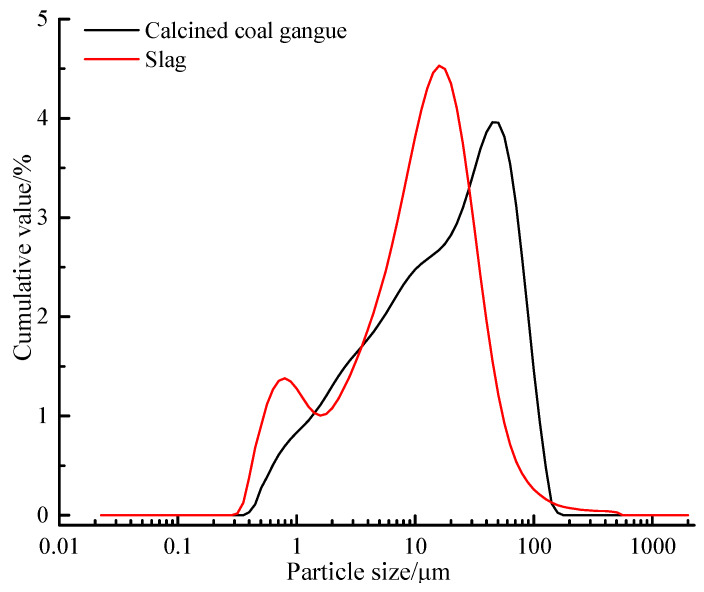
Calcined coal gangue and slag particle size analysis.

In this study, we chose high-alumina-clay rock coal gangue from Shanxi, and the coal gangue powder used in the test was calcined at 700 °C for 2 h in a muffle furnace. The slag is an S95 grade granulated blast furnace slag from Hebei Iron and Steel Plant. Table 1 shows the chemical composition of the calcined coal gangue and slag. It can be seen clearly that the calcined coal gangue belongs to the silica-alumina material, while the slag belongs to the calcium-silicon-alumina material. The D8-ADVANCE X-ray powder diffractometer from Bruker, Germany was used to testing the calcined coal gangue and slag, with a scanning speed = 2°/min and step size = 0.02°, continuously scanning specimens at 10° to 70° (2θ°). Figure 1 is the XRD patterns of coal gangue and slag powders; it can be seen that the main mineral components of the calcined coal gangue are quartz and muscovite, while the main mineral components of the slag are calcium-aluminum yellow feldspar and calcium-magnesium yellow feldspar. The particle size distribution of the calcined coal gangue and slag materials is shown in Figure 2, and the median particle sizes of the two materials were 17.252 μm and 10.529 μm, respectively. In this paper, 96% sodium hydroxide (NH) and sodium silicate (NS) solution (26.5% SiO_2_, 8.5% Na_2_O, 65% H_2_O, modulus 3.22) were selected as alkali activators.

Table 2 shows the mix proportion of AACGS mortar, and the liquid–solid ratio of the specimens is 0.46. There are a total of 10 groups of specimens, including two factors of different slag content and alkali activator modulus (the alkali activator modulus M is the ratio of the amount of Si_2_O substance in the alkali activator to the amount of Na_2_O substance). The configuration process of alkali activator modulus and the preparation and mixing process of mortar are referred to in [40]. The freshly mixed AACGS mortar was quickly poured into a trigeminy mortar mold of 40 mm × 40 mm × 160 mm, and the vibration table was shaken for the 60 s. The molds were covered with thin polyethylene films and cured for 24 h at relative humidity (RH) = 95% ± 1% and T = 20 ± 2 °C. The specimens were demolded, then transferred to standard curing room for curing to the test age.

### 3.2. Experimental Programme

#### 3.2.1. Compressive Strength

In order to determine the relationship between the fractal characteristics of the microstructure of AACGS mortar and the macro strength, The YAW-300 pressure tester was used to test the 28 d compressive strength of the AACGS mortar specimens, and the loading speed was 0.5–0.8 MPa/s. Three specimens were tested for each group, and the arithmetic mean of the three specimens was taken as the compressive strength.

#### 3.2.2. Pore Structure

The MIP test used the American AutoPore Ⅳ 9510 automatic mercury intrusion meter. After the AACGS mortar specimens were crushed and cored, the hydration was stopped with isopropanol, and then dried under vacuum at low temperature for 24 h. In order to ensure the accuracy of the test results, each group of test specimens was not less than 25 specimen particles (0.5 cm–1 cm). Continuous mercury injection was used, the contact angle and surface tension of mercury were 130° and 485 dynes/cm, respectively. The pore size distribution curve of the mortar specimen was obtained by changing the injection pressure. The pore volume, porosity, and pore size distribution curve of AACGS mortar specimens can be obtained by the MIP method. 

#### 3.2.3. Micro Morphology

The micro-morphology of AACGS mortar specimens was observed and analyzed using the Japanese SU8020 field emission scanning electron microscope. After the AACGS mortar specimens were crushed and cored, the hydration was stopped with isopropanol, and then dried under vacuum at low temperature for 24 h. The size of the AACGS mortar specimen used for the SEM test was approximately 1.5–2 cm. The specimen surfaces were sputter-coated with gold-palladium prior to imaging. In order to obtain high-quality SEM photos during the SEM test, an acceleration voltage of 15 kV was used [41].

## 4. Result and Discussion

### 4.1. Compressive Strength Analysis

**Figure 3:**
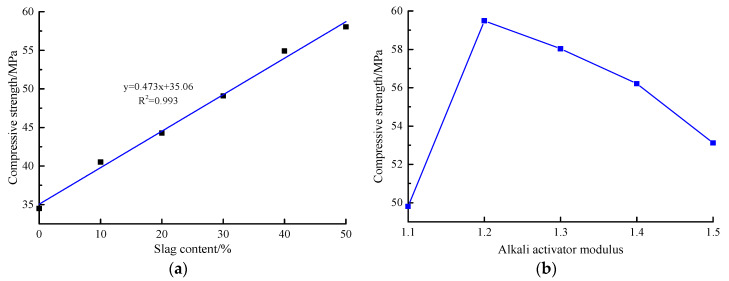
Compressive strength of alkali-activated coal gangue-slag (AACGS) mortar specimens. (**a**) Slag content; (**b**) Alkali activator modulus.

Compressive strength is the most important macroscopic index to evaluate advanced concrete materials. Figure 3 shows the compressive strength of the AACGS mortar specimens. Figure 3a shows the slag content factor. It can be clearly seen that with the slag content increases, the compressive strength of the AACGS mortar specimens increases, showing an excellent positive linear relationship. The pozzolanic activity of slag is higher than that of calcined coal gangues [13]. With the increase of slag content in the mixed mortar, the C–(A)–S–H gel production in AACGS mortar increased, making the matrix denser and thus increasing the compressive strength. Figure 3b shows the alkali activator modulus factor. It can be clearly seen that with the increase of alkali activator modulus, the compressive strength of the AACGS mortar increased first and then decreased. When the alkali activator modulus was 1.2, the compressive strength of the AACGS mortar specimen was the highest. Furthermore, when the modulus of the alkali activator was 1.2 and 1.3, there was no significant difference in compressive strength (difference of 1.46 MPa). In order to reduce the dosage of solid NH, the modulus of the alkali activator used in this paper was 1.3. Surprisingly, the AACGS mortar specimens had the lowest compressive strength when the alkali activator modulus was 1.1. The main reason was that the high alkali activator modulus needed to add more solid NH, and the higher OH^−^ concentration accelerated the depolymerization and polycondensation process of calcined coal gangue and slag, so that many coal gangue and slag powder did not participate in the geological polymerization reaction, and the residual filled in the specimen, thus reducing the compressive strength.

### 4.2. Fractal Characteristics and Fractal Dimension of AACGS Mortar Pores

**Figure 4:**
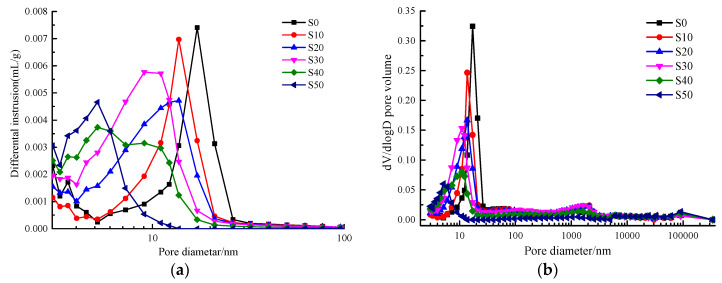
Mercury intrusion porosimetry (MIP) results of AACGS mortar with different slag content. (**a**) Differential pore size distribution of AACGS mortar within the range of 3–100 nm, and (**b**) differential pore volume versus pore size diameter.

The pore structure is an essential component of the microstructure of the mortar, and the pore structure characteristics affect the permeability and strength of the material. With advanced concrete materials for the preparation of mortar or concrete, the distribution of pore structure is more important than the effect of porosity on macroscopic performance [27]. In general, pore characteristics can be characterized by porosity, pore size distribution, and pore geometry (pore appearance and arrangement). When the porosity of different specimens is not much different, the critical index affecting the macroscopic properties of materials is pore geometry. Figure 4 shows the MIP results of AACGS mortar with different slag content. Figure 4a is the differential pore size distribution of AACGS mortar within the range of 3–100 nm; it can be clearly seen that there were significant differences in pore structure characteristics between the specimens. With the increase of slag content, the peak of the pore size distribution moved to the left, and the pore size distribution intensity of micropores or mesopores (d < 25 nm) increased. Figure 4b is a differential pore volume versus pore size diameter. The pore size distribution curve represents the threshold radius, which is located at the inflection point area behind the horizontal part. Below this radius, there was relatively little intrusion, and once it exceeded the threshold radius, the invasion began quickly [42]. With the increase of slag content, the pore size distribution moved to the left and the threshold radius increased. Furthermore, the steep slope in Figure 4b indicates the existence of a large number of pores, and it can be seen that the pore size structure became finer with increasing slag content.

**Figure 5:**
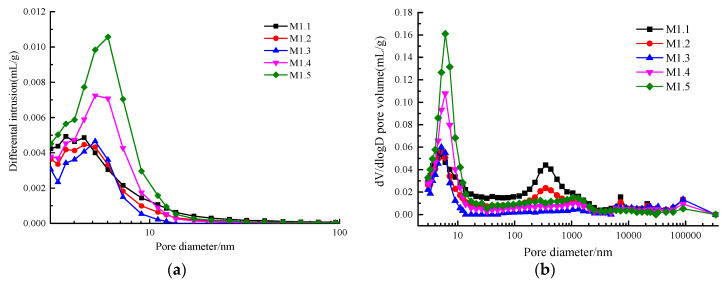
MIP results of AACGS mortar with different alkali activator modulus. (**a**) Differential pore size distribution of AACGS mortar within the range of 3–100 nm, and (**b**) differential pore volume versus pore size diameter.

Figure 5 shows the MIP results of AACGS mortars with different alkali activator modulus. Figure 5a is the differential pore size distribution of AACGS mortar within the range of 3–100 nm. It can be seen that the pore size corresponding to the peak of the pore distribution curve of each specimen had no significant difference (pore diameter is about 5 nm). With the increase of the alkali activator modulus, the peak height of the pore distribution curve shows a trend of decreasing first and then increasing. Figure 5b is the differential pore volume versus pore size diameter; with the increase of the alkali activator modulus, the pore size distribution curve did not move left and right, but the pore volume decreased first and then increased. When the modulus of alkali activator was 1.2 and 1.3, the microporous or mesoporous volume was lower.

The fractal dimension of pore volume in this paper is calculated according to Equation (7). The fractal dimension of the pore volume can be calculated by directly using the test data of MIP based on the change characteristics of pore volume and pore diameter. Table 3 is the pore fractal dimension and other pore characteristic parameters of the AACGS mortar specimens, including pore fractal dimension, porosity, total pore area, median pore dimension, and average pore dimension. The fractal dimension of pores characterizes the complexity of the spatial distribution pattern of the pores in the material. The larger the value of the fractal dimension of the pores, the more complicated the pore spatial distribution pattern of the specimen. The fractal dimension of the pores reflects the distribution state of the internal pore structure of the specimen to a certain extent, which is necessarily related to the macro-mechanical properties of the AACGS mortar specimens. 

**Figure 6:**
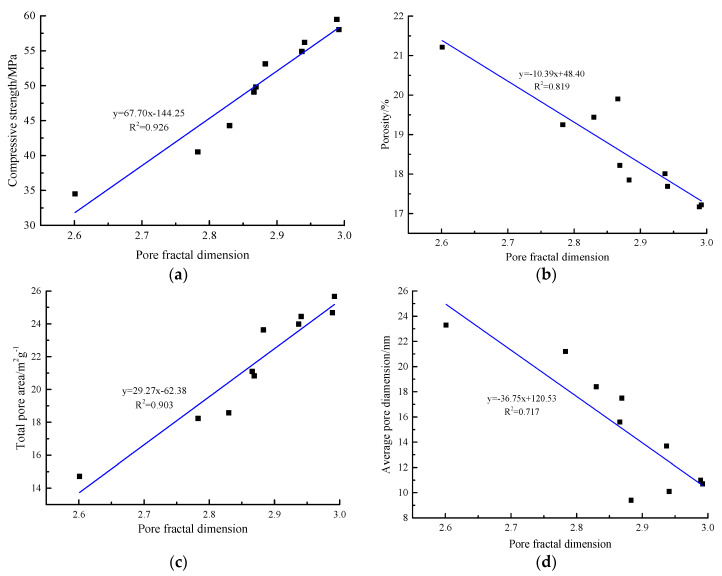
The relationship between the compressive strength and pore feature parameter of AACGS mortar specimens and the pore fractal dimension. (**a**) Compressive strength, (**b**) porosity, (**c**) total pore area, (**d**) average pore dimension.

Figure 6 shows the relationship between the compressive strength, pore characteristic parameters of the AACGS mortar specimens, and the pore fractal dimension, and Figure 6a shows the relationship between the compressive strength and the pore fractal dimension. It can be clearly seen that there was an excellent positive linear relationship between the compressive strength and the pore fractal dimension (regression coefficient R^2^ = 0.926). With the increase of the compressive strength of the AACGS mortar specimens, the pore fractal dimension of the specimens increased. The pore fractal dimension characterizes the complexity of the pore structure [43]. Figure 6b shows the relationship between the porosity and the pore fractal dimension, and it can be clearly seen that there was an excellent negative linear relationship between porosity and fractal dimension (regression coefficient R^2^ = 0.819). Figure 6c shows the relationship between the total pore area and the pore fractal dimension. There was an excellent positive linear relationship between the total pore area and the pore fractal dimension (regression coefficient R^2^ = 0.903). The larger the total surface area of the pores, the larger the volume of the smaller pores, the rougher and more complex the structure, and the larger the pore fractal dimension. Figure 6d shows the relationship between the average pore size and the pore fractal dimension. It can be clearly seen that there was a negative linear relationship between the average pore size and the pore fractal dimension (regression coefficient R^2^ = 0.717), and the correlation was the same as that of the porosity structure.

Active Ca in solid precursors has a significant impact on polymerization, and due to the admixture of slag, the main reaction products changed from N–A–S–H gels to C–A–S–H gels when the content of Ca increased. The larger the pore fractal dimension, the more complicated the pore structure, indicating that more C–(A)–S–H gels were produced or stacked in the AACGS mortar specimens [40], resulting in a decrease in the pore size of the mortar specimens. On the whole, the pore structure had a strong correlation with the pore fractal dimension, which can be used to predict the pore characteristic parameters (including porosity, total pore area, and average pore diameter, etc.) of the specimens by the pore fractal dimension.

### 4.3. Fractal Feature Analysis of SEM Photos

The SEM test is a commonly used detection method for analyzing the micro-morphology of mortar or concrete, but the photos obtained by the SEM test are grayscale images, and different grayscale image values constitute the texture characteristics of the images. The results obtained from SEM photos were qualitative analysis results, and the texture characteristics or complexity of the images could not characterize quantitatively. The fractal dimension can express the spatial information and texture information of the image. The larger the fractal dimension, the more complex the texture and rougher the structure of the SEM image, and vice versa. That is, the fractal dimension can be used to represent the texture complexity of the SEM image [16].

**Figure 7:**
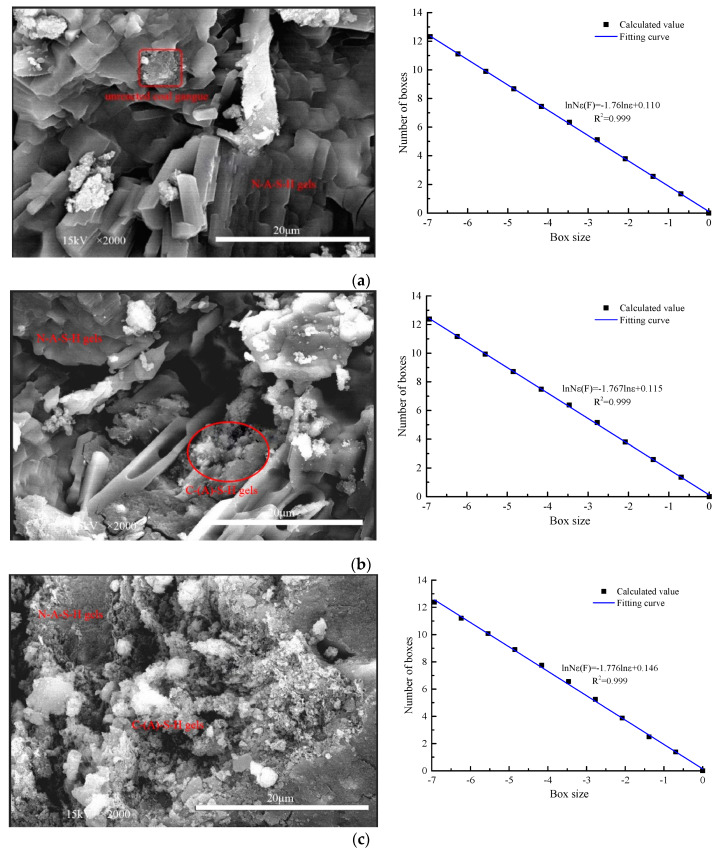

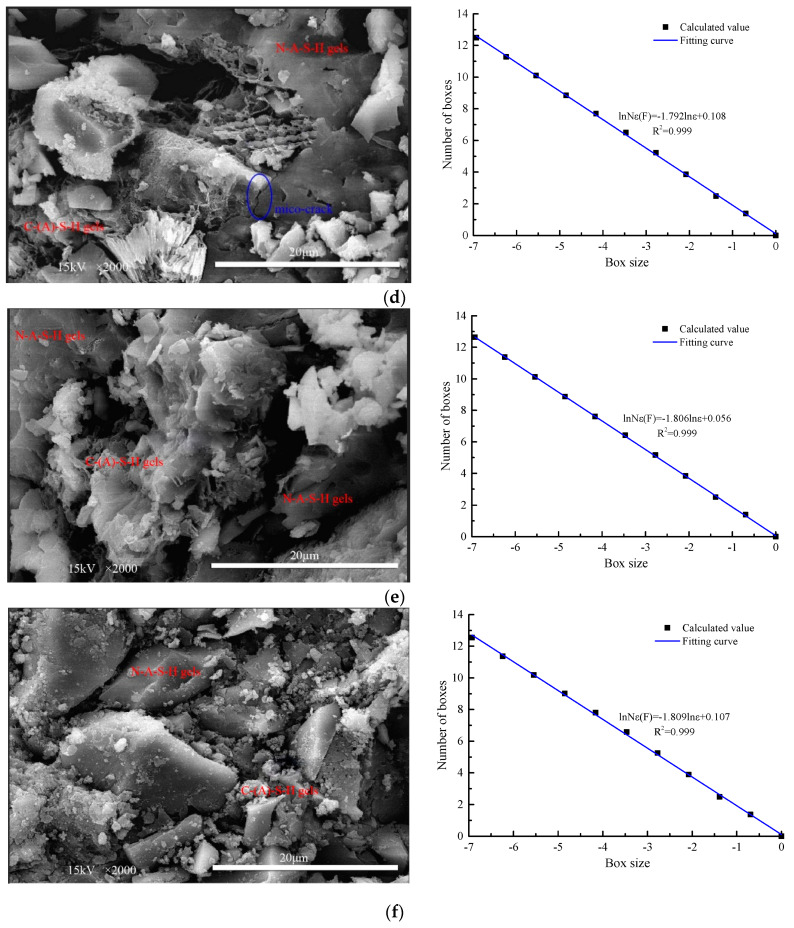
SEM photos and counting box dimension of AACGS mortar specimens (×2000). (**a**) S0, (**b**) S10, (**c**) S20, (**d**) S30, (**e**) S40, (**f**) S50.

The main hydration products of AACGS mortar include N–A–S–H gel, C–(A)–S–H gel, and other sodium alumino-silicate gels, all of which show a disordered network structure and have excellent compatibility [14]. The proportion and combination of the various hydration products directly affect the microstructure of AACGS mortar and then affect the macro mechanical properties of the mortar. Figure 7 shows the SEM photos and calculated box dimensions of the AACGS mortar specimens. It can be clearly seen from the SEM photos that the slag content had a significant effect on the surface morphology and structure of the AACGS mortar. With the increase of slag content, the hydration products of AACGS mortar produced more C–(A)–S–H gels, the flocculent hydration products gradually increased, and the structure of the hydration products became denser. 

**Figure 8:**
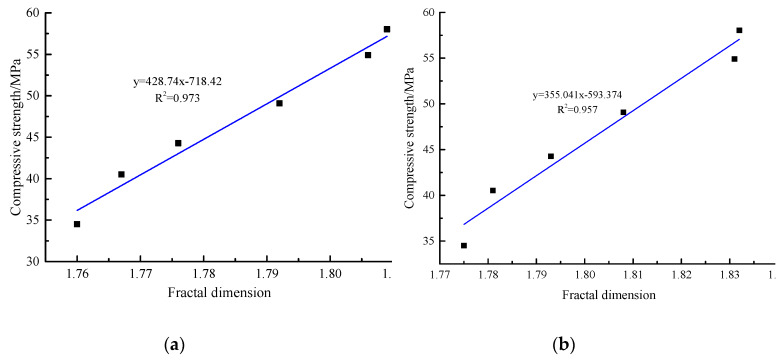
The relationship between the compressive strength of AACGS mortar and the fractal dimension. (**a**) ×2000, (**b**) ×1000.

According to the box dimension method mentioned in Section 2, the texture information contained in the limited area of SEM photos was quantitatively characterized. The binarization image of the SEM image can be obtained based on computer image processing and uses the box dimension method to calculate the fractal dimension of the binarization image. All SEM photos were selected at the same magnification and voltage. Table 4 shows the SEM photo fractal dimension of AACGS mortar. It can be found that with the increase of slag content, the larger the SEM photos fractal dimension was, which is related to the complexity of microstructure. Furthermore, the larger the magnification factor of SEM photo was, the smaller the fractal dimension value was. The SEM photos fractal dimension can quantitatively characterize the complexity of the microscopic hydration products on the surface of the specimen, and there must be a specific relationship between it and the macroscopic strength. Figure 8 shows the relationship between the compressive strength of AACGS mortar and the fractal dimension. It can be clearly seen that the fractal dimension values increased with the increase of slag content, and the compressive strength of AACGS mortar specimens increased with the increase of the fractal dimension, and the compressive strength and fractal dimension of AACGS mortar showed a significant positive linear relationship. Furthermore, they had regression coefficient R2 values of 0.973 and 0.957, respectively.

### 4.4. Discussion and Outlook

Both MIP method and the SEM method are commonly used to characterize advanced concrete materials. MIP data can directly obtain the total porosity and pore curve distribution of the specimens. The SEM photos are the most commonly used method to characterize the microscopic morphology of the specimens, but its photos cannot provide quantitative data. In this paper, based on the volume fractal model and the box dimension method, the fractal dimensions of the pore structure and the SEM photos are calculated respectively. Both fractal dimensions have a good linear relationship with the compressive strength.

The pore structure of different materials is completely different from the SEM image; that is, the calculated fractal dimension is also different. The fractal dimension of the pore structure can be calculated directly from the MIP data, and the MIP data are not accidental. Therefore, the fractal dimension of the pore structure is studied more. A large number of researchers have shown that the pore structure of concrete has obvious fractal characteristics [44,45,46]. Fu and Yu [47] calculated the fractal dimension of the pore structure of aerated concrete blocks and found that there was a significant linear relationship between pore fractal dimension and pore structure characteristics. Based on the MIP method and the fractal model, Jin et al. [43] obtained the relationship between the fractal dimension of the pore surface of cement mortar and the pore characteristic parameters, which is the same as the rule of this paper.

However, the fractal dimension of SEM photos first needs to be processed by binarization image with the help of the computer, and then the fractal dimension is calculated according to the box dimension method. SEM fractal dimension is related to photo quality, and its affecting factors include material type, SEM equipment, magnification, etc. Table 4 shows the effect of magnification on the SEM fractal dimension, and the larger the magnification of the SEM photos, the smaller the fractal dimension. As can be seen in Figure 8, the fractal dimension of the SEM photos shows a good positive correlation with the compressive strength under the same magnification. When studying the changes in the microstructure of different materials, researchers generally use SEM photos of the same magnification for comparison. The quality of SEM photos taken by different SEM equipment is different. However, in the actual test, the researchers often use the same SEM equipment for tests. Therefore, the errors caused by SEM photo quality factors can be ignored. The studies of SEM photos fractal dimension have not been wide, and there is a lack of relevant data and research on whether different material types also show the same rule. Risovic et al. [37] compared the fractal dimension of aluminum foil surface using SEM image and electrochemical impedance spectroscopy, and found that there was a significant positive linear relationship between SEM image fractal dimension and electrochemical impedance spectroscopy, and the fractal dimension values of all specimens had a good correlation. With the same material system and the same equipment, the fractal dimension of SEM photos with the same magnification is calculated, and the results are comparable. In future research, the effect of different material systems on the fractal dimension of SEM photos needs to experiment and discussed by more researchers.

## 5. Conclusions

In this paper, the relationship between the micro fractal dimension of AACGS mortar and the macro strength and pore characteristic parameters were studied. The following concluding remarks can be drawn:

(1). When the slag content is 0–50%, the compressive strength of AACGS mortar specimens increases with the increase of the slag content, and the slag content shows a significant positive linear relationship with the compressive strength.
(2). The pore fractal dimension and compressive strength show a significant positive linear relationship, and the pore structure and pore fractal dimension have a strong correlation, through the pore fractal dimension can predict the pore characteristic parameters of the specimens. The pore fractal dimension has a strong positive linear relationship with the total pore area and has an excellent negative linear relationship with porosity and average pore dimension.
(3). The binarization image of the SEM image can be obtained based on computer image processing and uses the box dimension method to calculate the fractal dimension of the binarization image. With the increase of slag content, the fractal dimension of SEM photos of AACGS mortar specimens increased, and the compressive strength of AACGS mortar and fractal dimension showed a significant positive linear relationship.
(4). The pore fractal model and the box dimension method were introduced into the AACGS mortar. Due to the difference between the two theories, the fractal dimension of the pore structure was quite different from the fractal dimension of SEM photos. However, both fractal dimensions could be used separately for the prediction of macroscopic performance. This study provides a basis for the application of fractal theory in the field of alkali-activated materials.
(5). Different from the fractal dimension of pore structure, SEM photos have a contingency, which is affected by material type, SEM equipment, and magnification. When the same researcher calculates the fractal dimension of SEM photos, SEM equipment and magnification can be ignored. However, whether different material systems show the same variation rules still needs to be studied and discussed by research scholars together.

## Figures and Tables

**Table 1:** Chemical compositions of calcined coal gangue and slag /wt%.

Parameters	SiO_2_	Al_2_O_3_	CaO	Fe_2_O_3_	MgO	Na_2_O	SO_3_	TiO_2_	LOI
Coal gangue	56.56	36.78	0.62	1.95	0.22	0.42	0.03	2.10	1.32
Slag	30.58	14.04	38.43	0.35	10.57	0.57	2.36	1.93	1.17

**Table 2:** Mix proportion of AACGS mortar.

Specimens	Coal Gangue/g	Slag/g	Standard Sand/g	NH	NS	Additional H_2_O	Alkali-Activator Modulus
S0	1000	0	2750	94.17	580.85	188.35	1.3
S10	900	100	2750	94.17	580.85	188.35	1.3
S20	800	200	2750	94.17	580.85	188.35	1.3
S30	700	300	2750	94.17	580.85	188.35	1.3
S40	600	400	2750	94.17	580.85	188.35	1.3
S50	500	500	2750	94.17	580.85	188.35	1.3
M1.5	500	500	2750	77.70	617.30	168.36	1.5
M1.4	500	500	2750	85.60	599.82	177.94	1.4
M1.2	500	500	2750	103.50	560.18	199.68	1.2
M1.1	500	500	2750	113.71	537.57	212.08	1.1

**Table 3:** AACGS mortar specimen pore fractal dimension and other pore characteristic parameters.

Specimens	Pore Fractal Dimension	Porosity/%	Total Pore Area/m^2^g^−1^	Median Pore Dimension/nm	Average pore Dimension/nm
S0	2.601	21.21	14.710	21.9	23.3
S10	2.783	19.25	18.229	18.3	21.2
S20	2.830	19.44	18.575	17.7	18.4
S30	2.866	19.90	21.094	15.3	15.6
S40	2.937	18.01	23.977	13.9	13.7
S50	2.992	17.22	25.665	11.6	10.7
M1.5	2.883	17.85	23.630	8.5	9.4
M1.4	2.941	17.69	24.446	9.3	10.1
M1.2	2.989	17.17	24.677	12.9	11
M1.1	2.869	18.22	20.828	97.3	17.5

**Table 4:** The SEM photos fractal dimension of AACGS mortars.

Magnification	S0	S10	S20	S30	S40	S50
×2000	1.76	1.767	1.776	1.792	1.806	1.809
×1000	1.775	1.781	1.793	1.808	1.831	1.832
